# Editorial: Biomechanics of aging: advances in exercise and intervention strategies for older adult wellness

**DOI:** 10.3389/fpubh.2026.1843143

**Published:** 2026-05-11

**Authors:** Bojan Masanovic, Predrag Bozic, Selcuk Akpinar, Milos Petrovic, Nejc Sarabon

**Affiliations:** 1 Faculty for Sport and Physical Education, University of Montenegro, Niksic, Montenegro; 2 Western Balkan Sport Innovation Lab, Podgorica, Montenegro; 3 Serbian Institute of Sport and Sports Medicine (SISSM), Belgrade, Serbia; 4 Physical Education and Sports Department, Nevşehir Haci Bektaş Veli University, Nevşehir, Türkiye; 5 Faculty of Health Promotion, Sport and Leisure Studies, University of Iceland, Reykjavik, Iceland; 6 Faculty of Health Sciences, University of Primorska, Izola, Slovenia

**Keywords:** aging biomechanics, exercise innovations, healthy longevity, older adults, physical activity interventions, public health, quality of life

## 1 Introduction

The fields of biomechanics and gerontology have significantly advanced our understanding of physiological alterations accompanying human aging. Aging is characterized by progressive declines affecting various systems, including the nervous system, muscle-tendon units, and joint and bone structures, which collectively lead to impairments in physical health, motor control deficits, and increased risk of functional limitations (1). These age-related structural and functional deteriorations, initially subtle, can gradually progress from mild functional impairment to a state of significant incapacity and even prolonged bed rest (2). While such changes are an inherent and inevitable component of biological aging, growing evidence demonstrates that their trajectory can be modified—delayed, attenuated, or partially prevented—through timely and targeted interventions (3).

However, the implications of these age-related biomechanical changes extend far beyond the individual level. With global life expectancy continually rising, the proportion of the world's population above 60 years is projected to increase dramatically, with estimates suggesting that older adults may constitute nearly 60% of the population in certain regions in the coming decades (4). This profound demographic shift will inevitably amplify the societal and healthcare burden associated with age-related functional decline. Consequently, the need to preserve functional independence, mobility, and overall wellbeing in older adults is not merely a clinical concern but an urgent public health priority.

Recent research underscores the effectiveness of non-pharmacological approaches—particularly physical exercise, nutrition, and technology-driven interventions—in mitigating these biomechanical and physiological consequences of aging (3, 5, 6).

This Research Topic aims to consolidate current knowledge and emerging evidence regarding the biomechanics of aging and the effectiveness of exercise as well as other targeted non-pharmacological interventions for promoting physical functionality and overall wellbeing among older adults. Therefore, this Research Topic was designed to consolidate current knowledge on the biomechanics of aging and to advance understanding of the effectiveness of exercise and other targeted non-pharmacological interventions in preserving and improving biomechanical function and overall wellbeing in older adults. Its aim is to provide evidence-based practical and theoretical insights that can contribute to scientific advancement, optimization of intervention strategies, and the development of healthcare and public health policies supporting healthy and active aging.

## 2 Contribution to the field

This Research Topic seeks to consolidate and expand a growing body of new knowledge on the biomechanics of aging and to present advances in exercise and intervention strategies aimed at enhancing functional capacity and wellbeing in older adults. The 25 studies published within this Research Topic—comprising 12 original research articles (eight cross-sectional studies, one qualitative descriptive study, two randomized controlled trials, and one prospective cohort study) and 13 review articles (eleven systematic reviews and meta-analyses, one systematic review, and one scoping review)—offer readers a valuable opportunity to expand their knowledge in this field. The original research articles included a total of 44,300 older adults aged 60 to 90+ years, as well as 16 young adults (<35 years) and 14 fitness staff members from six countries (United Kingdom, China, Taiwan, Turkey, the United States, and South Korea). In addition, the 13 review studies synthesized evidence from 175 primary studies involving a total of 12,223 participants across 27 countries (China, the United States, Australia, India, South Korea, Italy, Japan, Canada, Germany, Norway, Denmark, Sweden, Iran, Finland, Spain, France, the United Kingdom, Greece, Belgium, Pakistan, Ecuador, Thailand, Taiwan, Brazil, Portugal, Singapore, and Turkey), with participant ages ranging from 46 to 88 years.

The studies included in this Research Topic reflect a wide range of methodological approaches, thematic perspectives, and study populations. This diversity offered several possible ways to organize and interpret the contributions. For the purposes of this editorial, we grouped the papers into three broad thematic categories: (1) Review studies on non-pharmacological interventions, (2) Observational studies on lifestyle and health in aging, and (3) Intervention-based studies.

The first group consisted of 13 review studies (10 systematic reviews with meta-analyses, one systematic review, one meta-analysis, and one scoping review). All of these studies synthesize existing evidence on the effects of various non-pharmacological interventions aimed at improving health, functional capacity, and quality of life in older adults. Their findings highlight: the positive impact of exercise and therapeutic movement on older adults' health, including improvements in pain and joint function, apathy, balance and fall prevention, physical function, and muscle strength in individuals with different health conditions (Pi et al.; Jia et al.; Zhong et al.; Deng et al.; Ma et al.); the beneficial role of movement-based interventions in the management of specific diseases and aging-related syndromes, where approaches such as physical exercise, mind–body exercise, resistance training, and combined regenerative and exercise therapies improve vitality, social functioning, mental health, body composition, muscle strength, gait speed, and overall functional outcomes (Xi et al.; Chu et al.; Zeng et al.; Oka et al.); and the effectiveness of more recent or complex intervention approaches—including multicomponent programs, virtual reality interventions, digital health interventions, and digitalized traditional Chinese exercises—in improving functional parameters, reducing fall risk, enhancing cognitive performance (e.g., attention, processing speed, and executive function), and improving muscle mass and strength (Kasicki et al.; Chen, Sun et al.; Chen, Yao et al.; Tu et al.).

The second group consisted of eight cross-sectional studies examining associations between behavior, health, functional capacity, and risk factors in older adults. Their findings indicate: significant age-related sensorimotor decline and the importance of the combination of cognitive impairment and slow gait as a marker for identifying older individuals at high risk of multimorbidity (Alvarez-Hidalgo et al.; Zhu et al.); the beneficial role of regular walking, physical activity, and sport participation in improving health outcomes in older adults, including optimizing cognitive function, improving sleep, and attenuating the negative impact of low HDL cholesterol and chronic conditions such as hypertension and diabetes (Li, Chen et al.; Cai et al.; Cho et al.; Zheng et al.); the dual role of internet use in aging populations, where low to moderate use may support physical and mental health through digital inclusion and intergenerational interaction, while excessive use may negatively affect health by reducing physical activity (Li, Cai et al.; Zheng et al.); and finally, that counting daily steps represents a simple and practical way for older adults to monitor whether they achieve recommended levels of physical activity (Chen, Chen et al.).

The third group consisted of two randomized controlled trials, one prospective cohort study, and one qualitative descriptive study, examining the effects of exercise or rehabilitation programs on the health of older adults or addressing their implementation in practice. Their results showed: that these programs (resistance training and Taiji Stick exercise) improved functional capacity, respiratory muscle strength, diaphragm thickness, lower limb strength, and dynamic balance, maintained upper limb strength, and reduced fall-related risks (Akkuş Uçar et al.; Cao et al.); that pulmonary rehabilitation provides clinically meaningful benefits for older adults with chronic obstructive pulmonary disease (COPD) (Feng et al.); and that resistance exercise in retirement communities is recognized for its potential to support at-risk residents (Liu et al.).

## 3 Conclusion

Taken together, the studies included in this Research Topic further strengthen the growing body of evidence on the importance of exercise and other non-pharmacological strategies in addressing the biomechanical and functional consequences of aging. The findings presented across the 25 contributions highlight the multifaceted role of physical activity and related interventions in preserving mobility, improving functional capacity, supporting cognitive and mental health, and reducing the risk of age-related functional decline.

Beyond confirming previously established benefits of exercise for healthy aging (7, 8), this Research Topic provides a broad spectrum of evidence-based insights and practical approaches. These include structured exercise programs, rehabilitation strategies, technology-assisted interventions, and simple monitoring tools such as step-counting approaches that can help older adults maintain recommended levels of physical activity. Together, these approaches contribute not only to improving physical and functional outcomes but also to supporting autonomy, independence, and overall quality of life in later life.

Importantly, many of the interventions and assessment approaches discussed in this Research Topic have already demonstrated practical applicability, offering clinicians, practitioners, and policymakers valuable guidance for promoting active and healthy aging. By integrating advances in biomechanics, exercise science, and public health perspectives, these studies help bridge the gap between scientific knowledge and real-world implementation.

Ultimately, we hope that the findings and perspectives presented in this Research Topic will stimulate further interdisciplinary research and inspire the development of innovative intervention strategies aimed at preserving functional capacity, enhancing wellbeing, and supporting healthy longevity in aging populations.
